# Comparative genomics of the plant-growth promoting bacterium *Sphingobium* sp*.* strain AEW4 isolated from the rhizosphere of the beachgrass *Ammophila breviligulata*

**DOI:** 10.1186/s12864-022-08738-8

**Published:** 2022-07-13

**Authors:** Brianna L. Boss, Abanoub E. Wanees, Shari J. Zaslow, Tyler G. Normile, Javier A. Izquierdo

**Affiliations:** Department of Biology, Hofstra University, Hempstead, NY 11549 USA

**Keywords:** PGPR, Rhizobacterium, *Sphingobium*, Beachgrass, Comparative genomics

## Abstract

**Background:**

The genus *Sphingobium* within the class Alpha-proteobacteria contains a small number of plant-growth promoting rhizobacteria (PGPR), although it is mostly comprised of organisms that play an important role in biodegradation and bioremediation in sediments and sandy soils. A *Sphingobium* sp. isolate was obtained from the rhizosphere of the beachgrass *Ammophila breviligulata* with a variety of plant growth-promoting properties and designated as *Sphingobium* sp. strain AEW4.

**Results:**

Analysis of the 16S rRNA gene as well as full genome nucleotide and amino acid identities revealed that this isolate is most similar to *Sphingobium xenophagum* and *Sphingobium hydrophobicum*. Comparative genomics analyses indicate that the genome of strain AEW4 contains unique features that explain its relationship with a plant host as a PGPR, including pathways involved in monosaccharide utilization, fermentation pathways, iron sequestration, and resistance to osmotic stress. Many of these unique features are not broadly distributed across the genus. In addition, pathways involved in the metabolism of salicylate and catechol, phenyl acetate degradation, and DNA repair were also identified in this organism but not in most closely related organisms.

**Conclusion:**

The genome of *Sphingobium* sp. strain AEW4 contains a number of distinctive features that are crucial to explain its role as a plant-growth promoting rhizobacterium, and comparative genomics analyses support its classification as a relevant *Sphingobium* strain involved in plant growth promotion of beachgrass and other plants.

**Supplementary Information:**

The online version contains supplementary material available at 10.1186/s12864-022-08738-8.

## Background

The genus *Sphingobium* within the Alpha-proteobacteria class contains strictly aerobic, chemo-organotrophic, Gram-negative, rod-shaped bacteria [1]. This genus largely consists of a variety of environmental isolates with a role in bioremediation and the biodegradation of pollutants. However, a number of plant-associated *Sphingobium* species have been recently isolated from the roots of various plants, as is the case of *Sphingobium endophyticus* obtained from the forest poppy *Hylomecon japonica* [2] and *Sphingobium rhizovicinum*, which was obtained from the rhizosphere of *Fortunella hindsii* [3]. Other species, including *Sphingobium chlorophenolicum*, have been observed to enhance their biodegradative capabilities in the presence of plants [4].

Beyond the genus *Sphingobium*, a large number of *Sphingomonadaceae* have been broadly identified to be plant-growth promoting rhizobacteria (PGPR) in the genus *Sphingomonas*, *Novosphingobium* and *Sphingopyxis*. Within the genus *Sphingomonas*, a variety of strains have been reported to have the ability to protect plants from pathogenic *Pseudomonas* species [1]. *Sphingomonas paucimobilis* ZJSH1 has been successfully used as a bioinoculant for the plant *Dendrobium officinale* leading to an increase in aboveground and belowground plant biomass [5]. *Sphingomonas panaciterrae* has been isolated from a ginseng field and observed to produce the plant hormone indole-3-acetic acid (IAA) [6]. *Novosphingobium pokkalii*, which was isolated from a coastal saline-tolerant rice variety, utilizes a number of plant exudates as energy sources, produces phytobeneficial compounds like acetoin, siderophores, and IAA, and increases root and shoot length, as well as root abundance in vitro [7]. *Novosphingobium* strain sp. SW9 has been identified as a nitrogen fixer and was shown to increase root and shoot length in wild rice compared to uninoculated controls [8]. There is less evidence of the potential plant growth-promoting abilities of the genus *Sphingopyxis*, although there have been reports of endophytic *Sphingopyxis* strains. *Sphingopyxis granuli* has been found in the leaves of *Oryza sativa*, and an IAA-producing strain of *Sphingopyxis* sp. was isolated from strawberry tissue [9, 10]. Therefore, previous literature demonstrate that the family *Sphingomonadaceae* contain numerous endophytes with a variety of plant-growth promoting properties.

The American beachgrass, *Ammophila breviligulata*, is a grass from the *Poaceae* family native to the Northeast and mid-Atlantic coast. It is considered to be an ‘engineer species’ due to its ability to stabilize sand dunes in barrier islands and coastal environments [11, 12]. Given that it inhabits an environment with high levels of solar irradiation, high salinity, and strong winds, *Ammophila breviligulata* exhibits extreme resistance to a variety of environmental stressors, while also displaying extensive growth in nutrient-depleted environments. However, very little is known about the plant-growth promoting bacteria that may be associated with this plant and its success growing in such a challenging environment. In this study, we describe the isolation of *Sphingobium* sp. strain AEW4 from the rhizosphere of *Ammophila breviligulata*, and propose some of the mechanisms it uses in its interaction with beachgrass as a PGPR, using sequence-based and genome-scale analyses supported by phenotypic analyses.

## Results and discussion

### Properties of rhizosphere isolates

**Fig. 1:**
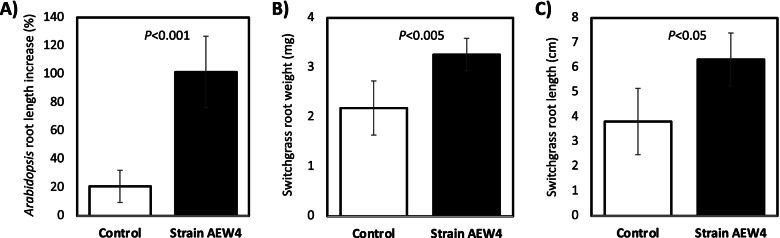
Plant-growth promotion assays using Arabidopsis and switchgrass inoculated with *Sphingobium* sp. strain AEW4 or uninoculated controls. Changes in root biomass were measured as increase in length for *Arabidopsis* seedlings (**A**), and final root weight (**B**) and length (**C**) in switchgrass. Graphed data represent mean ± SD of five biological replicates

A total of 87 isolates were obtained from rhizosphere soils attached to the roots of *Ammophila breviligulata*, which were consistently transferable to Schatz media as pure cultures. Of these, 32 isolates produced IAA at concentrations higher than 100 μg/mL (See Additional File 1) with high levels of representation by the genus *Sphingobium*, *Pseudomonas*, *Burkholderia*, *Paenibacillus, Mucilaginibacter* and *Azospirillum*. *Sphingobium* sp. strain AEW4 produced the highest concentration of IAA among these 32 isolates (497.3 ± 52.3 μg/mL), although other 3 other *Sphingobium* isolates produced between 340.2 ± 87.2 and 403.7 ± 1.7 μg/mL IAA. Strain AEW4 grew on Schatz media and LB media as distinct yellow-orange colonies, about 1-2 mm in diameter. Gram staining of strain AEW4 revealed it was a Gram-negative short rod. Strain AEW4 also produced evident zones of clearing when grown on Chrome Azurol S (CAS) agar media, pointing to its ability to sequester iron. In plant-growth promotion assays using *Arabidopsis* and switchgrass seedlings, strain AEW4 was shown to increase root biomass compared to uninoculated controls (Fig. 1). *Arabidopsis* roots inoculated with strain AEW4 grew an average of 101.5 ± 32.3% longer than the starting length, compared to the roots in the control group which only grew an average of 20.9 ± 11.3% longer (*P* < 0.001) than the initial length (Fig. 1A). In switchgrass grown from seed, root mass increased by 49.3% (*P* < 0.005, Fig. 1B) and root length increased by 65.6% (*P* < 0.05), Fig. 1C) in seeds inoculated with strain AEW4 when compared to uninoculated controls. Strain AEW4 was chosen for further study based on its ability to produce IAA and siderophores, as well as its PGPR ability in plant-growth promoting assays.

### Phylogeny of *sphingobium* sp. strain AEW4

**Fig. 2:**
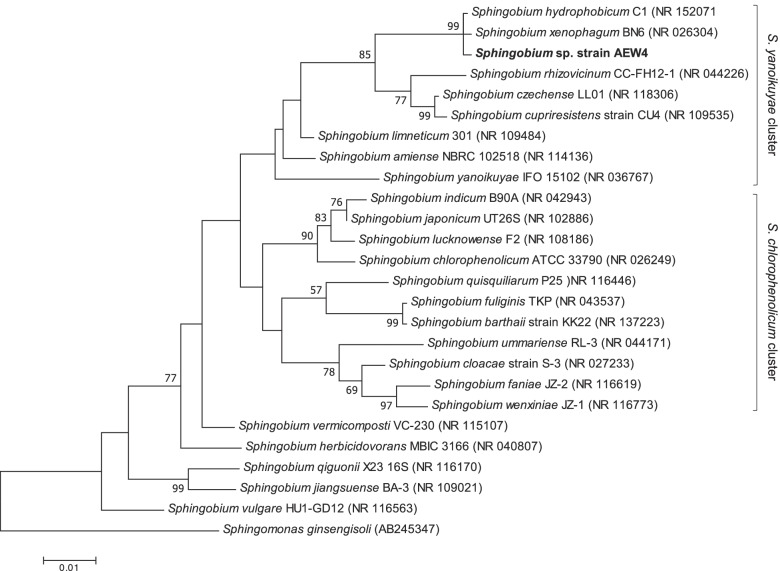
Phylogenetic tree of *Sphingobium* sp. strain AEW4 and closely related *Sphingobium* species. *Sphingomonas ginsensoli* was used as an outgroup. The maximum-likelihood tree was constructed using the MEGAX software with an alignment of 1494 nucleotides and bootstrapping (*n* = 1000)

The 16S rRNA gene sequence of strain AEW4 was sequenced in its entirety and displayed the highest sequence similarity to *Sphingobium xenophagum* (99.86%), followed by *Sphingobium hydrophobicum* (99.78%), and *Sphingobium czechenese* (97.34%). Phylogenetic analysis of the 16S rRNA gene of strain AEW4 is consistent with this finding and places this strain in a clade with *S. xenophagum* and *S. hydrophobicum*, as well as *S. czechenese* and *S. rhizovicinum*, other documented plant-associated *Sphingobium* species (Fig. 2). Of the initial *Sphingobium* species recognized and classified within this genus [1], the 16S rRNA gene of strain AEW4 and its most similar sequences all grouped within the cluster of *Sphingobium yanoikuyae*.

### Genomic features

*Sphingobium* sp. strain AEW4 has one chromosome containing 4,678,518 base pairs and a G + C content of 62.64%. The genome contains 4,255 protein coding sequences (95.55%), 17 regulatory and miscellaneous features (0.37%), and 59 RNA genes (1.28%). Out of the 4,525 coding sequences, 3,375 (73.35%) were assigned a function, while the remaining 1,150 (24.99%) were identified as hypothetical proteins or domains of unknown function. The genome sequence of *Sphingobium* sp. strain AEW4 has been deposited in GenBank under the accession number PYGL00000000.

**Fig. 3:**
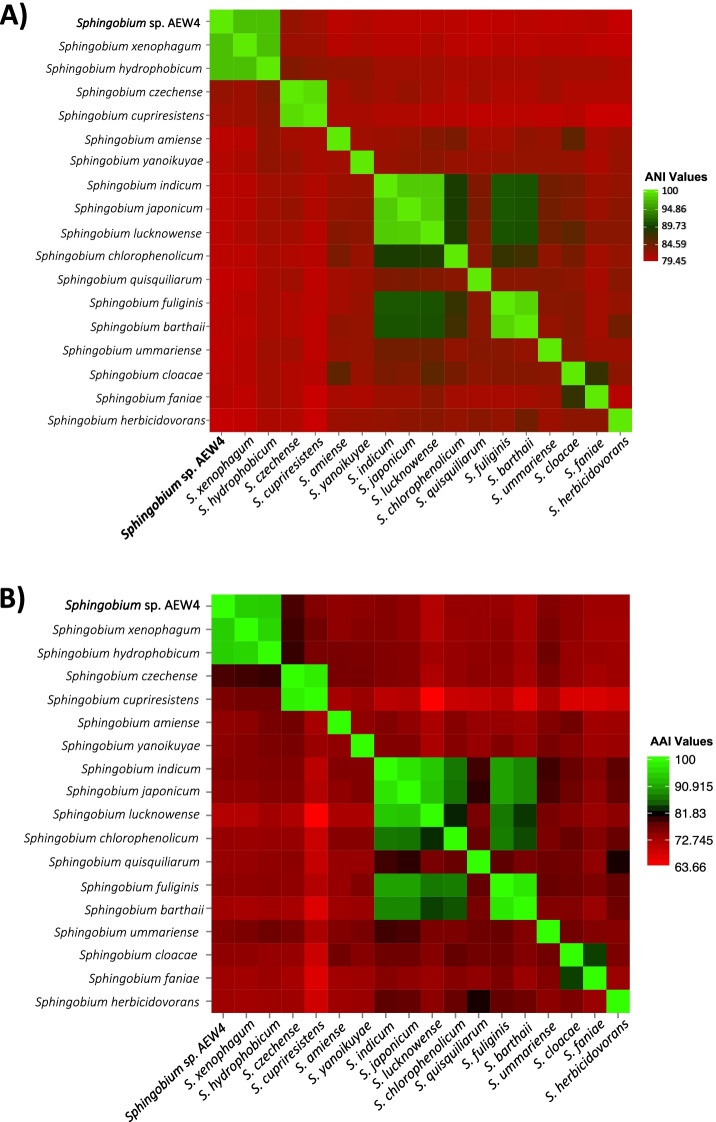
Average Nucleotide Identity (ANI) and Average Amino Acid Identity (AAI) of *Sphingobium* species. Heat maps show the variation in A) Average Nucleotide Identity (ANI) and B) Average Amino Acid Identity (AAI) for *Sphingobium sp*. strain AEW4 and 17 different species in the genus *Sphingobium*. The values scale shows percent similarity based on nucleotides, with red representing a low identity and green representing a high identity

In order to further investigate the phylogenetic relationship between strain AEW4 and within the *Sphingobium* genus, we performed a genome-wide comparison of strain AEW4 and 17 genomes of the *Sphingobium* species with highest 16S rRNA gene similarity using average nucleotide identity (ANI) and average amino acid identity (AAI). In both the ANI (Fig. 3A) and AAI (Fig. 3B) comparisons, *Sphingobium* sp. AEW4 shares the highest index similarity with its two most closely related organisms, *S. xenophagum* and *S. hydrophobicum*. Strain AEW4 shares 97.53% (ANI) and 94.59% (AAI) similarity with *S. xenophagum*, and 97.49% (ANI) and 94.54% (AAI) similarity with *S. hydrophobicum*, confirming the higher degree of similarity with *S. xenophagum* that was identified through 16S rRNA gene sequencing. When examining other groups of closely related species, we observed similar ANI and AAI values. For example, in the ANI analysis (Fig. 3A, Additional File 2), *Sphingobium indicum* shares 98.15% similarity with *Sphingobium japonicum* and 98.09% similarity with *Sphingobium lucknowense*. In the AAI analysis (Fig. 3B, Additional File 2), the grouping of *S. inducum*, *S. japonicum*, and *S. lucknowense* is also very similar with *S. indicum* sharing 96.97% similarity with *S. japonicum* and 94.1% similarity with *S. lucknowense*.

### Comparison of orthologs and unique genes

**Fig. 4:**
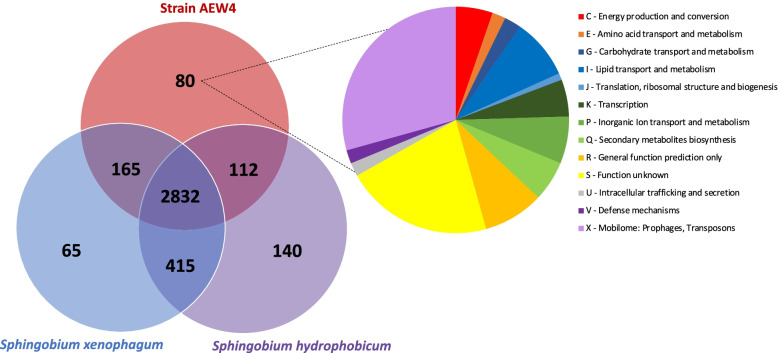
Genome-wide comparison of orthologous clusters between strain AEW4 and its two closest relatives. Proportions of shared and unique clusters in comparisons with *Sphingobium xenophagum* and *Sphingobium hydrophobicum* are shown in the Venn diagram*,* based on OrthoVenn analysis. Strain AEW4 contains 80 unique clusters when compared to these two species, with 208 proteins which are classified in the pie chart based on their functional role

The 4,525 protein-coding genes present in the genome of strain AEW were grouped into 3,809 orthologous clusters (Fig. 4). We performed a comparison of shared and unique orthologs between strain AEW4 and its two closest relatives, *Sphingobium xenophagum* and *Sphingobium hydrophobicum*, which revealed that 2832 orthologous clusters were shared between the three genomes. *S. hydrophobicum* shares 112 clusters with AEW4 compared to 165 clusters shared between AEW4 and *S. xenophagum*. However, *S. xenophagum* and *S. hydrophobicum* seem to have the highest amount of overlap, with 415 shared orthologous clusters. Strain AEW4 has 80 unique clusters out of a total of 3,809 when compared to its two closest relatives (Additional File 3). Aside from mobile elements and hypothetical proteins, many of the 208 genes identified in these unique clusters had GO annotations that included lipid transport and metabolism (8.7%), ferrichrome iron receptors, siderophores and heavy metal resistance genes (6.7%), secondary metabolite biosynthesis (5.8%), carbohydrate transport and metabolism (2.4%) and type IV secretion system proteins (1.9%) (Fig. 4, Additional File 3). Of these 208 genes, 97 (46.6%) had top BLASTp matches in other *Sphingobium* species, while 74 (35.6%) had matches in other genera from the *Sphingomonadaceae* like *Sphingomonas*, *Novosphingobium*, *Sphingopyxis* and *Sphingosinithalassobacter*. A total of 23 genes (11.1%) had top BLASTp matches in other Alpha-proteobacteria that were not *Sphingomonadaceae*, with the genus *Rhizoabdus*, *Rhizobium* and *Croceibacterium* having multiple matches. The remaining 15 genes (7.2%) had top matches in the Gamma- and Beta-proteobacteria, as well as the Firmicutes, Planctomycetes and Acidobacteria.

**Fig. 5:**
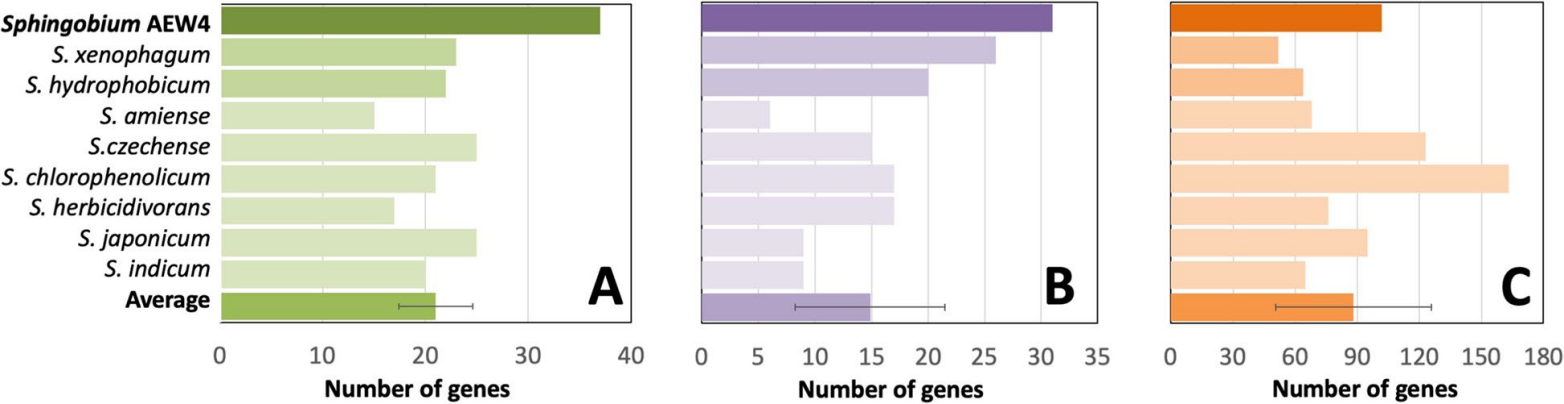
Comparison of key functional gene categories in *Sphingobium* sp. strain AEW4 with the other members of the genus ***Sphingobium.*** Numbers of genes are shown for monosaccharide metabolism (**A**), osmotic stress (**B**), and fermentation (**C**) for *Sphingobium* sp. strain AEW4, its two closest relatives, *S. xenophagum* and *S. hydrophobicum,* and other genomes within the genus, including an average ± SD for these nine members

In addition, functional gene categories annotated by Rapid Annotations using Subsystems Technology (RAST) were compared side by side with *S. xenophagum* and *S. hydrophobicum*, as well as 6 other relevant *Sphingobium* genomes. Strain AEW4 was found to have a larger number of genes than *S. xenophagum* and *S. hydrophobicum* in three important categories relevant to plant-associated microbes: carbohydrate utilization, osmotic stress and fermentation pathways (Fig. 5).

### Carbohydrate utilization

**Fig. 6:**
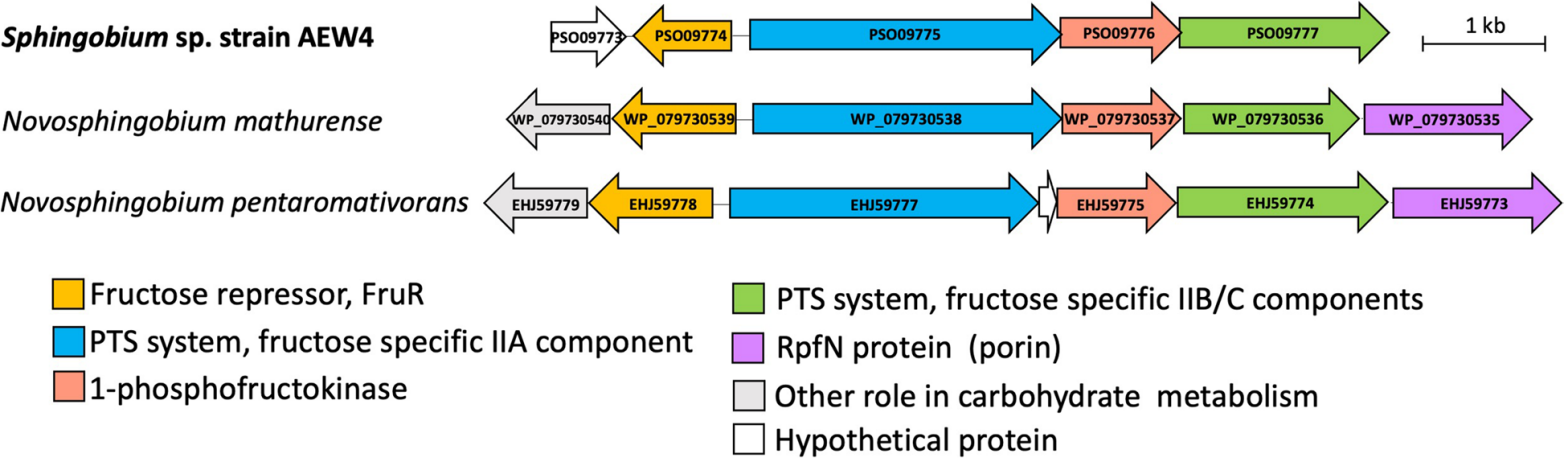
Organization of the fructose operon present in *Sphingobium* sp. strain AEW4. Unique genes identified in the genome of strain AEW4 are shown and compared to closest gene matches in operons of *Novosphingobium mathurense* and *Novosphingobium pentaromativorans* US6-1

Compared to other closely related genomes, *Sphingobium* sp. strain AEW4 has the largest number of genes dedicated to monosaccharide metabolism (Fig. 5A), with a total of 37 genes in this category, compared to 23 in *S. xenophagum*, 18 in *S. hydrophobicum*, and an average of 20 for closely related genomes of *Sphingobium* species. The genome of strain AEW4 has ten genes dedicated to fructose metabolism and one gene involved in mannose metabolism that are not found in either the *S. xenophagum* genome or the *S. hydrophobicum* genome, organized in an operon potentially responsible for fructose utilization (Fig. 6). The operon includes five genes, including a hypothetical protein (PSO09773), a phosphoenolpyruvate-protein phosphotransferase of the PTS system or IIA component (PSO09775) (EC 2.7.3.9), 1-phosphofructokinase (PSO09776) (EC 2.7.1.56), a fructose-specific IIB/C component of the PTS system (PSO09777) (EC 2.7.1.69), and a putative carbohydrate porin (PSO09794). A fructose repressor, FruR, (PSO09774) is located upstream of the operon, although it is transcribed in the opposite direction. Interestingly, this operon is found in strain AEW4 but not in its two closest relatives, *S. xenophagum* and *S. hydrophobicum*, or in any members of the *Sphingobium* genus, making it unique to strain AEW4 within its genus. Similar operons were found in *Novosphingobium pentaromativorans* and *Novosphingobium mathurense*, both within the *Sphingomonadaceae* family, but not closely related to strain AEW4 (Fig. 6). It should be noted that in both of these organisms, a carbohydrate-specific porin is located immediately downstream of the gene coding for the PTS system IIB/C component, whereas in strain AEW4 it is located a further downstream (PSO09794).

**Fig. 7:**
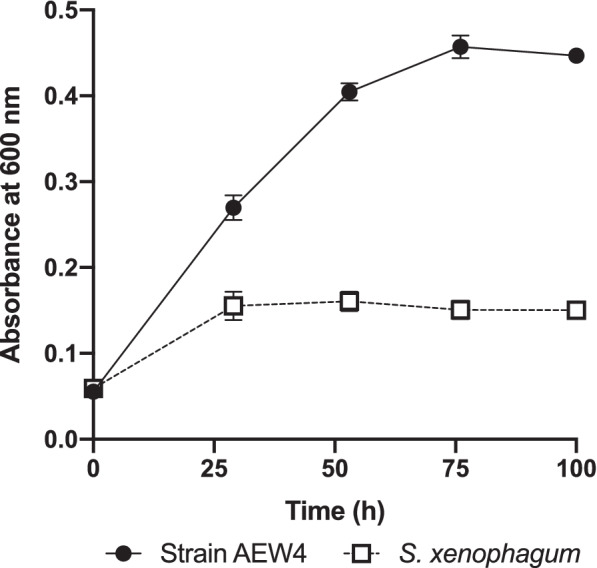
Fructose growth dynamics of *Sphingobium* species. Growth of *Sphingobium* sp. AEW4 and *Sphingobium xenophagum* on fructose as the sole carbon source. Growth dynamics data represent the mean ± SD between three biological replicates

To verify the ability of strain AEW4 to utilize this operon, we tested its ability to grow on fructose as the sole carbon source compared to *S. xenophagum* (Fig. 7). When both strains were transferred to media with fructose as the sole carbon source, strain AEW4 was able to grow fourfold compared to *S. xenophagum*, reaching an OD_600_ of 0.457 after 76 h of growth at 30 °C. Aside from the fructose operon, an endoglucanase (EC 3.2.1.4) that plays a role in mannose utilization has also been identified. The ability to utilize different monohydrates than the species most closely related to strain AEW4 may be of considerable use since this organism was isolated from rhizosphere soils, where sugars such as fructose and mannose are common [13], and where it could play a role in establishing a relationship between *Sphingobium* sp. strain AEW4 and its origin plant, *Ammophila breviligulata* [14].

In addition, there are multiple copies of genes involved in D-galacturonate and D-glucuronate utilization present in strain AEW4 that are only present in single copies in *S. hydrophobicum*, and completely missing in the *S. xenophagum* genome. These genes include 2-dehydro-3-deoxygluconate kinase (EC 2.7.1.45) (PSO10628, PSO11203), two copies of endo-1,4-beta-xylanase A precursor (EC 3.2.1.8) (PSO10884, PSO10833), D-mannonate oxidoreductase (EC 1.1.1.57) (PSO10629), a hexuronate transporter (PSO10587), and a uronate isomerase (EC 5.3.1.12) (PSO10599). Overall, these data suggest that strain AEW4 is equipped to process a broader variety of carbohydrates than *S. xenophagum* and *S. hydrophobicum*.

### Osmotic stress

Microorganisms that inhabit a coastal dune rhizosphere are challenged with a dynamic environment, with a wide range of osmotic stress present in the rhizosphere at any given time. The osmolarity of the rhizosphere has been observed to surpass that of the surrounding bulk soil [15, 16]. As microbes in the rhizosphere are unable to actively transport water in and out of their cells, they produce and accumulate intracellular solutes, known as compatible solutes, to prevent dehydration or cell rupture without interfering with normal enzymatic activity [15, 17, 18].

Although there are no unique genes involved in osmotic stress in strain AEW4, its genome has 31 genes associated with osmotic stress compared to 26 in *S. xenophagum*, 25 in *S. hydrophobicum,* and compared to an average of 15 in the genomes of six other closely related sequenced *Sphingobium* genomes (Fig. 5B). Strain AEW4 has a larger number of outer membrane proteins (3 in total) involved in osmoregulation, as well as more choline dehydrogenase genes (5 total) and choline sulfatase genes (7 total) than *S. xenophagum* and *S. hydrophobicum*. The genome also contains both a sarcosine oxidase complex and an ectoine biosynthesis operon, whereas the genomes of *S. xenophagum* and *S. hydrophobicum* only contain either a sarcosine oxidase complex or an ectoine biosynthesis operon, respectively.

Ectoine, one of the many compatible solutes, has been shown to provide osmoprotection to multiple species [17, 19], stimulate growth of rhizobacteria [20], and protect rhizobacteria against heat stress [21]. Unlike other compatible solutes, ectoine is not accumulated within cells but instead catabolized, resulting in the accumulation of other compatible solutes such as trehalose and glutamate [22, 23]. There are three genes essential for ectoine production in bacteria, which can be found in the *ectABC* operon [24]. The ectoine biosynthesis operon in strain AEW4 includes L-2,4-diaminobutyric acid acetyltransferase (*ectA*) (EC 2.3.1.178) (PSO10533), diaminobutyrate-pyruvate (DABA) aminotransferase (*ectB*) (EC 2.6.1.76) (PSO10546), L-ectoine synthase (*ectC*) (EC 4.2.1.108) (PSO10532), ectoine hydroxylase (*ectD*) (EC 1.14.11.55) (PSO10531), an aspartokinase (PSO10530), and MarR family transcriptional regulatory protein (EctR) (PSO10534). The presence of an aspartokinase and ectoine hydroxylase in addition to a complete *etcABC* operon has been linked to enhanced halotolerance in some methanotrophs [25]. This operon also includes a couple of transporter proteins expected to be associated with osmoprotection: a transmembrane transport protein (PSO10529) and proline/glycine transporter protein ProP (PSO10528).

Choline is the precursor of glycine betaine, which serves as an osmoprotectant. The genome of strain AEW4 contains a high-affinity choline uptake protein BetT (PSO10139), multiple choline dehydrogenases (EC 1.1.99.1) (PSO11054, PSO11121, PSO12832, PSO12909, PSO11866), and a choline-sulfatase (EC 3.1.6.6) (PSO09885). Choline dehydrogenases convert exogenous choline to betaine aldehyde, which is further metabolized to the osmoprotectant glycine betaine by a betaine aldehyde dehydrogenase (EC 1.2.1.8) [26]. There are also two betaine aldehyde dehydrogenase genes (PSO09838, PSO11057) that share 99.8% and 100% amino acid sequence similarity to corresponding genes in *Sphingobium hydrophobicum* (ASY46126, ASY45857), respectively. Choline-sulfatases catalyze the conversion of choline-O-sulfate and phosphorylcholine into choline. Choline and choline-O-sulfate have been known to act as osmoprotectants in many microbes, such as *Bacillus subtilis* [27] and *E. coli* [28]. However, in the case of *Sinorhizobium meliloti*, choline only serves as an osmoprotectant after subsequent oxidation to betaine and upon accumulation in the cell [26, 29].

The high level of osmotic stress genes in strain AEW4 is reflective of its habitat, a coastal dune rhizosphere, where drought and fluctuating salinity levels are the standard. The compatible solutes that accumulate in sand dune plant and microbe species are most likely in response to water stress. During high temperature seasonal temperatures, water deficits are known to develop in dune grass species like *Ammophila arenaria*, which produces high amounts of betaine as a response [30]. There is also evidence that compatible solutes, such as glycine betaine, can increase the heat stability of enzymes in heat-stressed leaves of dune plant species, as temperatures rise during the summer months [31].

### Other unique pathways in strain AEW4

A unique pathway involved in the metabolism of salicylate and catechol was found in the genome of strain AEW4. Salicylate is an anti-inflammatory compound found ubiquitously in plants, and produced by numerous bacteria, that has been shown to induce systemic acquired resistance (SAR) in plants [32], in addition to being related to siderophore activity in PGPR [33]. Bacterial salicylate production is known to proceed through the chorismate and isochorismate pathways [33, 34]. *Sphingobium* strain sp. AEW4 is able to convert isochorismate to salicylate with an isochorismate pyruvate lyase (PSO11389). Strain AEW4 has enzymes to metabolize both salicylate and catechol, with salicylate hydroxylase (EC 1.14.13.1) (PSO10593) and catechol 2,3-dioxygenase (EC 1.13.11.2) (PSO10594) genes, respectively. The enzyme salicylate hydroxylase is responsible for breaking down salicylate to catechol, and has been shown to have antifungal and antibacterial properties [35, 36]. Catechol can further be broken down by catechol 2,3-dioxygenases, which play a key role in the degradation of aromatic pollutants such as polycyclic aromatic hydrocarbons (PAHs) and benzene, toluene, ethylbenzene xylene (BTEX) compounds [37]. Neither gene is found in the two closest relatives, *S. xenophagum* and *S. hydrophobicum*, making them unique to strain AEW4 among these organisms. The two genes are located adjacent to each other in a larger operon which contains a hydrolase (PSO10595), a hypothetical cupin protein (PSO10596), and a fumarylacetoacetate hydrolase family protein (PSO10597), none of which are found in *S. xenophagum* or *S. hydrophobicum*.

The genome of strain AEW4 also revealed an uncommon catabolic pathway used to degrade phenylacetate, which is not present in the genomes of *S. xenophagum* or *S. hydrophobicum*. This phenylacetate degradation pathway is a central component of the phenylacetyl-CoA catabolon, which integrates multiple peripheral catabolic pathways that convert aromatic compounds such as tropic acid, phenylalanine, and lignin-related compounds to the common intermediate phenylacetate-CoA [38]. Many environmental pollutants such as styrene and ethylbenzene also pass through this degradation pathway [39, 40]. Currently, this is the only known bacterial pathway for degradation of phenylacetate, and it exists in approximately one sixth of all currently sequenced bacterial genomes. This unconventional hybrid pathway employs elements of aerobic and anaerobic ring metabolism, ensuring the degradation process can proceed in fluctuating oxygen conditions [41]. The 14 *paa* genes are responsible for phenylacetate catabolism and are organized into two transcriptional units: *paaZ* and *paaABCDEFGHIJK* [30, 42]. The *paa* operon in strain AEW4 consists of a *paaZ* (PSO10016), *paaB* (PSO10017), *paaC* (PSO10018), *paaD* (PSO10019), *paaE* (PSO10020), *paaG* (PSO10021), *paaH* (PSO10022), *paaI* (PSO10023), *paaJ* (PSO10024), *paaK* (PSO10025), and *paaA* (PSO10052) in consecutive order. Some of the operon follows the arrangement of other operons in the literature. For example, the genes in *paaGHIJK* code for a ring hydroxylating complex and all five genes are generally found adjacent to each other [30]. However, *paaF* (PSO10035), a phenylacetate-coA ligase, does not follow the usual arrangement in this operon in AEW4 (*paaABCDEF*), instead lying a few genes downstream. Similarly, there also appears to be an additional *paaI* gene located much further upstream in the genome (PSO10853).

Finally, an operon not broadly distributed within the genus *Sphingobium* involved in DNA repair has been found in strain AEW4. It consists of a DNA helicase (PSO09809), an SbcCD exonuclease Mre11 (PSO09810), and a Rad50 ATPase (PSO09811). The complex known as SbcCD is essential for the detection and repair of DNA double stranded breaks. Double-stranded breaks can be introduced during regular genomic upkeep or through environmental mutagens such as toxins or high frequency radiation [43]. Rad50 is an ATP binding protein and Mre11 possesses 3′5’ exonuclease activity. One of the main roles of this bacterial complex is to process DNA hairpins that arise at locations of inverted repeats [44, 45]. This operon is not found in *S. xenophagum* or *S. hydrophobicum*, and may be associated with strain AEW4 residing in the rhizosphere soils in sand dunes that may become exposed to high levels of ultraviolet radiation.

### Secondary metabolites

Using the antiSMASH platform we were able to identify four categories of secondary metabolites produced by strain AEW4. Among them, there is an operon for bacteriocin production (PSO11796, PSO11798, PSO09521), which includes two genes not found in *S. xenophagum* or *S. hydrophobicum*. There were also four genes involved in terpene production (PSO13755, PSO13756, PSO13582, PSO13583) found in all three organisms, strain AEW4, *S. xenophagum* and *S. hydrophobicum*. The antiSMASH platform also detected the pathway for ectoine production previously discussed, and a type III polyketide synthase (T3PKS) cluster (PSO11556, PSO11561, PSO11565, PSO11677, PSO11568, PSO11575, PSO11576, PSO11577, PSO11679, PSO11680).

### Heavy metal resistance

Heavy metals are commonly found in the environment in the form of pesticides, fertilizers, and industrial byproducts. Certain heavy metals are essential for basic microbial function, as they serve as cofactors for many proteins and enzymes, aid in maintaining osmotic balance, and play important roles in regulating gene expression [46, 47]. Heavy metals may also be required as essential nutrients for bacteria at nanomolar concentrations. At higher concentrations, they become toxic and can interfere with active formations of biological molecules, displacing metal ions and blocking essential functional groups [47, 48]. The genome of strain AEW4 contains multiple genes involved in heavy metal resistance, including resistance against copper, cobalt, cadmium, zinc, arsenic, and chromate. The genome of strain AEW4 contains two separate but identical systems for copper resistance, consisting of CopB (PSO11053, PSO10414), CopC (PSO11048, PSO10408), CopG (PSO10417), CopD (PSO11129, PSO10465), and multicopper oxidases (PSO11083, PSO10413). There are also copper homeostasis proteins, including a copper-translocating P-type ATPase (PSO12191, PSO10406, PSO10425, PSO10439) and a copper-specific multidrug resistance transporter (PSO11098). An operon for arsenic resistance was also identified, including two arsenate reductase genes (PSO10387, PSO10388) and two arsenic resistance proteins ArsH (PSO10384) and Acr3 (PSO10386). Some of these genes have duplicates elsewhere in the genome (PSO13707, PSO10708), adjacent to an operon repressor (PSO13706). A chromate resistance protein, ChrB (PSO11305), and a chromate transport protein, ChrA (PSO11327), are also present in the genome. Genes involved in cobalt-cadmium-zinc resistance include two transcriptional regulators (PSO10426, PSO10405), CzcB family proteins classified as efflux transporters (PSO11126, PSO10151, PSO10420, PSO10430), CzcC family of resistance-nodulation-cell division (RND) efflux outer membrane proteins (PSO11125, PSO10150, PSO10466, PSO10429), cobalt, zinc, and cadmium efflux system membrane fusion proteins (PSO13410, PSO09690, PSO12233), resistance proteins CzcA/CusA (PSO11127, PSO10152, PSO10421, PSO10431), and CzcD (PSO10468, PSO10438). Additionally, this strain contains multiple RND efflux outer membrane lipoproteins including CmeA (PSO11242, PSO11927), CmeB (PSO11243, PSO11837), CmeC (PSO13250, PSO09623, PSO11244, PSO11836), as well as a tripartite multidrug resistance operon (PSO13749, PSO13511, PSO13510). The presence of these genes is in line with other species within the *Sphingobium* genus, which have been isolated from sites with high levels of heavy metal contamination, including strains of *S. xenophagum* [38] and *S. hydrophobicum* [49].

### Resistance to antibiotics

Antibiotic production is a common trait among PGPR. It allows rhizobacteria to serve as potential biocontrol agents and it is associated with the ability to act as an antagonist against plant and soil pathogens [50]. The genome of strain AEW4 contains several beta-lactamase proteins (PSO10653, PSO11399, PSO09599, PSO13552, PSO13085, PSO13088), as well as two RND efflux transporters associated with acriflavin resistance, ArcB (PSO13409) and an ABC transporter subunit (PSO12234). An operon for macrolide export also exists, including macrolide specific export protein MacA (PSO13251), ABC transporter ATP-binding protein MacB (PSO13252, PSO13253), and RND outer membrane lipoprotein CmeC (PSO13250). Not included in this operon are multiple other proteins that make up the RND efflux system including CmeC, CmeB, and CmeA.

### Nitrogen metabolism

Unlike some root-associated bacteria, *Sphingobium* sp. AEW4 does not have the capacity to fix nitrogen as it lacks the *nif* operon. However, it does contain genes required for assimilatory nitrate and nitrite reduction. There are three nitrate reductase genes (PSO10231, PSO12889, PSO11740), two nitrite reductase genes encoding for a small and large subunit (PSO10232, PSO10233), two nitrate/nitrite specific transporters (PSO10214, PSO10235), one ABC nitrate transporter (PSO10236), and a response regulator NasT (PSO10237). The genome of AEW4 also contains a nitrite-sensitive transcriptional repressor NsrR (PSO10802), which is associated with nitrosative stress. Nitrate and nitrite reduction was confirmed through positive results after a 3-day growth experiment of strain AEW4 on nitrate broth with a Durham tube.

### Pathways of plant hormone production

Indole-3-acetic acid is a signaling molecule that plays an important role in plant-growth promotion [51, 52]. There are several pathways through which IAA is produced via tryptophan [53]. Although there is no clear evidence of a complete pathway present in strain AEW4, the genome contains enzymes from both the indole-3-acetamide (IAM) pathway and the indole-3-pyruvate (IPyA) pathway. Two genes that are associated with tryptophan metabolism have been identified in strain AEW4, specifically responsible for the conversion of indolic compounds to IAA. One identified gene, an aliphatic amidase *amiE* (PSO12555) (EC 3.5.1.4) converts IAM to IAA. This enzyme is homologous to the second enzyme (*iaaH*) involved in the IAM pathway. However, the first step is catalyzed by tryptophan-2-monooxygenase (*iaaM*), which converts tryptophan to IAM [54] and it is not present in the genome of strain AEW4. In addition to the aliphatic amidase, there are 14 aldehyde dehydrogenases (EC1.2.1.3.) which convert indole-3-acetaldehyde to IAA. The conversion of indole-3-acetaldehyde to indoleacetate is present in three of the known IAA pathways, including the IPyA, the tryptophan side-chain oxidase (TSO), and the tryptamine (TAM) pathways [22]. However, the TSO pathway has only ever been reported in strains of *Pseudomonas fluorescens* [53, 55].

The volatile compounds acetoin and 2,3-butanediol, are commonly found in PGPR and have been demonstrated to stimulate plant growth and induce systemic resistance [56, 57]. The genome of AEW4 contains an acetolactate synthase (EC 2.2.1.6) (PSO11504, PSO09834, PSO11366), which consists of a large and small subunit. It converts pyruvate to acetolactate [58], the first step in producing acetoin and 2,3-butanediol from pyruvate. The next step in the pathway is the production of acetoin from acetolactate, which happens enzymatically by an acetolactate decarboxylase (EC 4.1.1.5), or non-enzymatically in which acetolactate is oxidized to diacetyl [59]. Diacetyl is converted to acetoin and 2,3-butanediol by acetoin reductase. The AEW4 genome does not contain an acetolactate decarboxylase gene. However, a 2-hydroxycyclohexanecarboxyl-CoA dehydrogenase (PSO13104) in the AEW4 genome could potentially function as an acetoin reductase, converting diacetyl to acetoin and 2,3-butanediol. Methyl Red-Voges Proskauer (MR-VP) tests conducted using strain AEW4 resulted in no acetoin or butanediol production, which confirms that this may be an incomplete pathway. Further testing will be necessary to determine other metabolites are produced by the genes in this pathway.

### Siderophores and iron uptake

Siderophores are small molecules produced by microbes that sequester iron from the environment [60]. *Sphingobium* sp. strain AEW4 tested positive for siderophore production on CAS agar media, and its genome contains a variety of genes involved in iron sequestration. There are 34 genes that encode for TonB receptors, 8 of which (PSO09750, PSO10031, PSO10196, PSO10263, PSO10845, PSO11515, PSO13079, PSO13125) were identified by the OrthoVenn analysis as unique to strain AEW4 and known to facilitate the transport of iron-bound siderophores into the cell [23]. It was also possible to identify 4 FecR family proteins (PSO09758, PSO10846, PSO11215, PSO11378) in the genome of strain AEW4, which have been reported to be involved in regulation of iron and siderophore uptake in *E. coli* [61]. Finally, the genome of strain AEW4A also contains genes for the ferrous iron uptake system, FeoAB (PSO12449, PSO12244), which has been identified to be required for iron uptake in *Bradyrhizobium japonicum*, a symbiotic rhizobacterium that does not produce siderophores [62].

## Conclusions

In the present work, we have described the isolation of strain AEW4, a plant growth promoting rhizobacterium (PGPR) in the genus *Sphingobium*, as well as the general features of its genome with a comparison to the genomes of its most closely related *Sphingobium* species, *S. xenophagum* and *S. hydrophobicum*. Our results strongly suggest that, although this strain is most closely related to *S. xenophagum*, it possesses many unique features that explain its positive interaction with the host plant resulting in increased biomass and growth observed in potted experiments and plant growth assays. Some of these unique features include the production of indole acetic acid (IAA), siderophore production pathways, carbohydrate utilization capabilities and adaptations to the coastal dune environment that include genes involved in osmotic stress resistance.

## Methods

### Isolation and characterization of strains

Samples of rhizosphere soil were collected from the roots of the American beachgrass *Ammophila breviligulata* along a 600 m-long transect in Cedar Beach, NY by collecting sand directly attached to the root. Javier Izquierdo and Shari Zaslow undertook the formal identification of the plant material used in the study and voucher specimens were deposited in the Microbiology Laboratory at Hofstra University. Rhizosphere soils samples were suspended in 0.1 M MgSO_4_ prior to plating. Suspensions were transferred to a modified Schatz agar medium [63] consisting of the following, in grams per liter: KH_2_PO_4_, 1.0 g; NH_4_NO_3_, 1.0 g; MgSO_4_•7H_2_O_,_ 0.2 g; FeSO_4_•7H_2_O, 0.001 g; CaCl_2_, 0.01 g; glucose, 1.04 g; sodium gluconate, 1.32 g; fructose, 1.08 g; succinate, 0.7 g; sucrose, 0.86 g; and agar, 15.0 g. Carbon sources were filter sterilized and added after media sterilization. All plates were incubated at 30 °C for 2 to 5 days and isolates were transferred to Schatz agar media for further isolation. All isolates were also grown on LB media and on CAS agar media [64] to test for the production of siderophores.

### Plant growth promotion

All isolates were screened for the production of indole-3-acetic acid (IAA) using liquid Schatz media amended with 1 g/L of L-tryptophan and allowing them to grow at 30 °C for 5 days. Cultures were vortexed and centrifuged for 5 min at 10,000 rpm. Culture supernatants were mixed with Salkowski reagent in a 5:1 ratio (v/v) and incubated at room temperature for 30 min. Optical density was measured and recorded at 490 nm, and compared to a standard curve for resulting IAA production [65].

In vivo plant-growth promotion was tested using seeds of *Arabidopsis thaliana* and switchgrass (*Panicum virgatum*). For each plant, seeds were vortexed in sterile deionized water (diH_2_O) for 5 s to prevent seeds from sticking together. Seeds were then subsequently washed in 95% ethanol for 5 min, 10% bleach solution for 5 min, and diH_2_O for 5 min. Washes were repeated four times and seeds were resuspended in diH_2_O after the final wash. *Arabidopsis thaliana* Col-0 seeds were obtained from Lehle Seeds (Round Rock, Texas) and placed at 4 °C, in the dark for 72 h. *Arabidopsis* seeds were grown on Murashige and Skoog media [66] and incubated in a growth chamber for 96 h at 22–23 °C with 12 h of light each day in a growth chamber. Initial measurements were then taken of the root and shoot length. The root of each seedling was submerged in either sterile Schatz media or Schatz media inoculated with strain AEW4 (10^8^ CFU/ml) for 10 min and then placed on fresh Murashige and Skoog media. The plates are wrapped in surgical tape, placed back in the growth chamber for 96 h, after which final root length measurements were taken for each. Switchgrass seeds were inoculated with either 1 mL of sterile Schatz media or 1 mL Schatz media inoculated with strain AEW4 (10^8^ CFU/ml) directly on top of the seed and planted in an autoclaved 50:50 sand:soil mixture in a small pot. They were then immediately inoculated with either 1 mL of sterile Schatz media or 1 mL Schatz media inoculated with strain AEW4 (10^8^ CFU/ml) directly on top of the seed. Potted plant experiments were performed in biological replicates of 5 and grown for 4 weeks in a greenhouse. After 4 weeks, roots were washed with tap water, and root length and mass were measured for all replicates. Statistical significance was assessed for each experiment through unpaired Student’s t-tests.

### Isolation of genomic DNA and 16S rRNA gene sequence analysis

Genomic DNA of *Sphingobium* sp. strain AEW4 was obtained using the GenElute genomic DNA isolation kit (Sigma Aldrich). 16S rRNA gene amplification and sequencing was performed using primers 8F (5'-AGAGTTTGATCCTGGCTCAG-3') and 1492R (5'-TACGGYTACTTGTTACGACTT-3'). Relevant similar sequences were identified through a BLASTN search [67]. Alignments of these sequences and phylogenetic trees were constructed with MEGAX using the maximum likelihood method. For tree construction, *Sphingomonas ginsengsoli* was used as an outgroup and 1000 bootstrap replicates were performed [68].

### Genome sequencing

An Illumina library was prepared using Nextera DNA Sample preparation kit (Illumina) following the manufacturer's user guide. The initial concentration of DNA was determined to be 28.2 ng/µl using the Qubit® dsDNA HS Assay Kit (Life Technologies). A total of 50 ng of DNA was used to prepare the library. The sample underwent simultaneous fragmentation and addition of adapter sequences during a limited-cycle (5 cycles) PCR in which unique indices were added to the sample. Following the library preparation, the final concentration of the library (15.1 ng/µl) was measured using the Qubit® dsDNA HS Assay Kit (Life Technologies), and an average library size of 776 bp was determined using the Agilent 2100 Bioanalyzer (Agilent Technologies). The library was then diluted to 10 pM and clustered using the cBot (Illumina) and sequenced paired end for 500 cycles using the HiSeq 2500 system (Illumina). Assemblies were created using SeqMan NGen from the Lasergene genomics package version 12.1.0 (DNAStar, Madison, WI). Annotation was conducted with the NCBI Prokaryotic Genome Annotation Pipeline [69] and with Rapid Annotations using Subsystems Technology (RAST) server version 2.0 [70].

### Comparative genomics and ortholog analysis

The sequenced genomes of the 17 *Sphingobium* species most closely related to isolate AEW4 were downloaded from GenBank, and were used to calculate Average Nucleotide Identity (ANI) and Average Amino Acid Identity (AAI) values with the Kostas Lab AAI and ANI calculators [71]. Pairwise ANI and AAI values were used in the generation of heat maps using Heatmapper [72]. Comparative genome-wide analysis of orthologous genes was performed with OrthoVenn [73] to compare the predicted protein sequences in strain AEW4 with those of two other closely related species, *Sphingobium xenophagum* DSM 6383 and *Sphingobium hydrophobicum*, both of which were downloaded from their respective public NCBI repositories. The predicted proteins of these three genomes were uploaded into the OrthoVenn web server for identification and comparison of orthologous clusters. An interactive Venn diagram, summary counts, and functional summaries of clusters shared between species were visualized using OrthoVenn. The putative function of each unique gene was determined using BLASTP.

### Fructose utilization, nitrate reduction and butanediol production assays

*Sphingobium* sp. strain AEW4 and *Sphingobium xenophagum* DSM 6383 (DSMZ) were grown on Schatz media with all carbon sources until they reached an OD_600_ of 0.4 and were then transferred to Schatz media with only fructose as the sole carbon source. The OD_600_ of these cultures was determined over a 96-h growth period in triplicate experiments. Nitrate and nitrite reduction were tested through inoculation of nitrate broth with a Durham tubes (BD, Franklin Lakes, NJ). Butanediol production was tested through inoculation of a Methyl Red-Voges Proskauer (MR-VP) to test for the production of the intermediate acetoin using MR-VP broth media (BD, Franklin Lakes, NJ).

## Supplementary Information

**Additional file 1. **Production of indole-3-acetic acid(IAA) by beachgrass rhizosphere isolates. Values are reported as averageand standard deviation from triplicate experiments.

**Additional file 2.**Average Nucleotide Identity (ANI) and Average Amino Acid Identity (AAI)values among selected*Sphingobium*species

**Additional file 3. **Complete list of unique proteins and clusters identified through OrthoVennanalysis comparing*Sphingobium* sp. strain AEW4 with *Sphingobiumxenophagum*and*Sphingobium hydrophobicum*.

## Data Availability

The whole genome data is publicly available in GenBank under the accession number PYGL00000000 (https://www.ncbi.nlm.nih.gov/nuccore/PYGL00000000).

## Abbreviations

AAI: Average Amino Acid Identity
ABC: ATP-binding cassette
ANI: Average Nucleotide Identity
BLAST: Basic Local Alignment Search Tool
BTEX: Benzene, toluene, ethylbenzene xylene
CAS: Chrome Azurol S
CFU: Colony forming units
GO: Gene Ontology
IAA: Indole-3-acetic acid
IAM: Indole-3-acetamide
IPyA: Indole-3-pyruvate
OD: Optical Density
PAH: Polycyclic aromatic hydrocarbons
PGPR: Plant-growth promoting rhizobacteria
PTS: Phosphotransferase system
RAST: Rapid Annotations using Subsystems Technology
RND: Resistance-nodulation-cell division
SAR: Systemic acquired resistance
TAM: Tryptamine
TSO: Tryptophan side-chain oxidase
T3PKS: Type III polyketide synthase
