# Supplementary figures and images for: Comparative genomics of the plant-growth promoting bacterium Sphingobium sp. strain AEW4 isolated from the rhizosphere of the beachgrass Ammophila breviligulata
Source: BMC Genomics. 2022 Jul 13;23:508. doi: 10.1186/s12864-022-08738-8 (PMC9281055; doi:10.1186/s12864-022-08738-8)

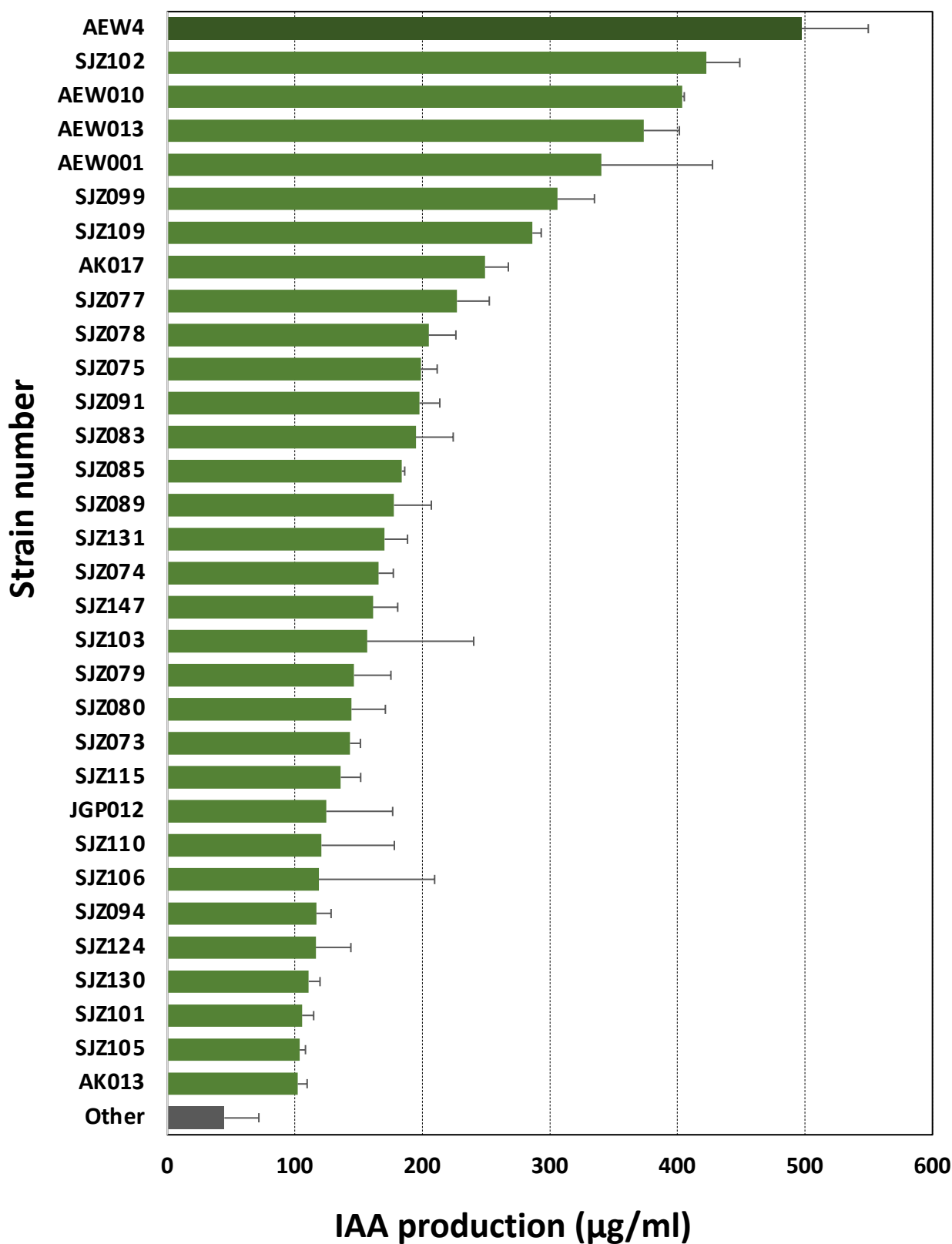

Supplement: Supplementary file 1 — Additional file 1. Production of indole-3-acetic acid(IAA) by beachgrass rhizosphere isolates. Values are reported as averageand standard deviation from triplicate experiments. [file 12864_2022_8738_MOESM1_ESM.pdf]
